# Beneficial Effects of Empagliflozin Are Mediated by Reduced Renal Inflammation and Oxidative Stress in Spontaneously Hypertensive Rats Expressing Human C-Reactive Protein

**DOI:** 10.3390/biomedicines10092066

**Published:** 2022-08-24

**Authors:** Hana Malínská, Martina Hüttl, Irena Marková, Denisa Miklánková, Silvie Hojná, František Papoušek, Jan Šilhavý, Petr Mlejnek, Josef Zicha, Jaroslav Hrdlička, Michal Pravenec, Ivana Vaněčková

**Affiliations:** 1 Institute for Clinical and Experimental Medicine, 14220 Prague, Czech Republic; 2 Institute of Physiology, Czech Academy of Sciences, 14220 Prague, Czech Republic

**Keywords:** SHR-CRP, SGLT-2 inhibitor, gene expression, age, lipid metabolism

## Abstract

Gliflozins (inhibitors of sodium-glucose cotransporter 2) show many beneficial actions beyond their antidiabetic effects. The underlying mechanisms of these additional protective effects are still not well understood, especially under non-diabetic conditions. Therefore, we analyzed the effects of empagliflozin in young (3-month-old) and adult (12-month-old) male spontaneously hypertensive rats (SHR) expressing human C-reactive protein (CRP) in the liver. SHR-CRP rats are a non-diabetic model of metabolic syndrome, inflammation, and organ damage. Empagliflozin was given in a daily dose of 10 mg/kg body weight for 8 weeks. Both age groups of SHR-CRP rats treated with empagliflozin had lower body weight, decreased weight of fat depots, reduced ectopic fat accumulation in the liver and kidneys, and decreased levels of plasma insulin and β-hydroxybutyrate. Empagliflozin effectively reduced ectopic renal fat accumulation, and was associated with decreased inflammation. Exclusively in young rats, decreased microalbuminuria after empagliflozin treatment was accompanied by attenuated oxidative stress. In adult animals, empagliflozin also improved left ventricle function. In conclusion, in young animals, the beneficial renoprotective effects of empagliflozin could be ascribed to reduced lipid deposition in the kidney and the attenuation of oxidative stress and inflammation. In contrast, hepatic lipid metabolism was ameliorated in adult rats.

## 1. Introduction

Diabetes is among the leading causes of mortality worldwide. As a chronic disease, it is associated with both micro- and macrovascular complications including coronary artery disease, stroke, diabetic nephropathy, neuropathy, and retinopathy. Traditional antidiabetic drugs, such as sulphonylureas or insulin, often have undesirable side effects, including hypoglycemia and weight gain; some of them potentially increase cardiovascular risk [1]. In contrast, new antidiabetics, in particular gliflozins, inhibitors of sodium-glucose cotransporter 2 (SGLT-2), show many beneficial actions beyond their antidiabetic effects. Their effects are mediated through the selective SGLT-2 inhibition at the renal proximal tubule, promoting glucose and sodium excretion, thus leading to significant improvement of glucose control together with the lowering of blood pressure and body weight [2]. The mechanisms underlying the reduction of cardiovascular events and renal protective effects are still poorly understood. Chronic low-grade inflammation is being increasingly recognized as a key feature associated with type 2 diabetes mellitus and its complications [3]. Experimental findings suggest that part of the renoprotective effects of SGLT-2 inhibition may be related to anti-inflammatory actions at the kidney level. The underlying mechanisms to explain this anti-inflammatory effect are multiple, and may involve body weight loss, reduction in adipose tissue inflammation, as well as decreased ectopic fat accumulation in the kidney or attenuation of oxidative stress.

Major large-scale clinical trials such as EMPA-REG, CANVAS, DECLAIRE-TIMI 58, and CREDENCE [4,5,6,7] demonstrated positive cardiovascular effects (decrease of the composite endpoints consisting of cardiovascular death, non-fatal myocardial infarction, and non-fatal stroke) of empagliflozin, canagliflozin and dapagliflozin in several thousand diabetic patients. Furthermore, in the DAPA-CKD trial, SGLT-2 inhibitors were also shown to offer renal protection in non-diabetic patients with proteinuria [8]. A recent systematic review and meta-analysis of the DAPA-HF and EMPEROR trials showed improvements in the composite renal endpoint regardless of the presence of diabetes or baseline estimated glomerular filtration rate [9]. Renal benefit has been attributed to metabolic and hemodynamic effects, including lowering of blood pressure, attenuation of glomerular hyperfiltration, body weight loss, and decreased plasma volume, as well as reduced renal hypoxia.

Systemic and renal inflammation is involved in the initiation and progression of diabetic and non-diabetic kidney disease. It has been previously demonstrated in diabetic animal models that the anti-inflammatory potential of SGLT-2 inhibitors is associated with decreased glomerular and tubulo-interstitial damage [10,11,12]. However, the underlying molecular mechanisms are not completely understood, and only limited information is available on SGLT-2 iinhibitor-mediated anti-inflammatory effects under experimental non-diabetic conditions. In fact, several studies demonstrated cardioprotective effects in experimental non-diabetic models [13,14]. The beneficial effects of gliflozins on renal function were observed in spontaneously hypertensive rats with heart failure [15] and also partly in rats after 5/6 nephrectomy [16]. Our studies in non-diabetic models, namely in hypertensive Ren-2 transgenic rats (TGR) [17], a model of angiotensin II-dependent hypertension, as well as in a model of metabolic syndrome and prediabetes—hereditary hypertriglyceridemic rats (HHTG) [18], showed the beneficial effects of empagliflozin treatment without a glucose-lowering effect. In these models, SGLT-2 inhibition led to a reduction in body weight and visceral adiposity, a decrease of insulin and leptin levels, and an improvement of hepatic metabolism, as well as the attenuation of oxidative stress and inflammation. There were also distinct effects depending on the model used, i.e., blood pressure decrease of TGR, and attenuation of oxidative stress and cell senescence in HHTG rats.

In the current study, we tested the metabolic and renoprotective effects of empagliflozin in spontaneously hypertensive rats expressing human C-reactive protein (SHR-CRP rats), a non-diabetic model of metabolic syndrome with severe hypertension, systemic inflammation, metabolic and hemodynamic disturbances, and target organ injury [19]. The expression of transgenic human CRP in these rats is associated with increased hepatic and renal oxidative tissue damage, increased plasma levels of interleukin 6, and a marked increase of microalbuminuria, which is accompanied by renal histopathologic changes such as fibrosis and inflammatory cellular infiltrates in the interstitium of the kidney [19]. To determine the mechanisms of empagliflozin beyond its antidiabetic effect, we focused our attention on its metabolic and renal effects in this non-diabetic model, both in young rats and in adult animals with the established systemic inflammation. In addition, we analyzed several genes involved in the inflammatory processes in the kidneys.

## 2. Materials and Methods

### 2.1. Animals

The SHR-CRP/OlaIpcv transgenic rats (referred to as SHR-CRP) were derived by microinjections of zygotes with a construct containing the cDNA for human CRP under control of the apolipoprotein E promoter with the objective of driving expression of the CRP transgene in the liver, where CRP is normally produced [19]. To investigate the effects of empagliflozin on the metabolic parameters and on kidney injury associated with human CRP, we randomized male SHR-CRP transgenic rats at the age of 3 months (young) and 12 months (adult) into two groups, with or without empagliflozin treatment. In each group, we studied 8 males. The rats were fed ad libitum a standard laboratory diet or the same diet containing empagliflozin in a daily dose of 10 mg/kg body weight for 8 weeks. The rats were housed in an air-conditioned animal facility. All experiments were performed in compliance with the Animal Protection Law of the Czech Republic and were approved by the Ethics Committee of the Institute of Physiology, Czech Academy of Sciences, Prague (Protocol Nr. 47/2019), and conformed to the European Convention on Animal Protection and Guidelines on Research Animal Use (Directive 2010/63/EU).

### 2.2. Metabolic Parameters in Epididymal Adipose Tissue and Myocardium

As a marker of visceral adipose tissue insulin sensitivity, basal and insulin-stimulated lipid syntheses were determined ex vivo in isolated epididymal fat pad by measuring the incorporation of ^14^C-U glucose into lipids, as described previously [20]. Basal and adrenaline-stimulated lipolysis in the epididymal adipose tissue were measured ex vivo and evaluated as the release of non-esterified fatty acids (NEFA). Glucose and palmitate oxidation in the myocardium were measured ex vivo in heart tissue sections determined as the incorporation of ^14^C-U glucose and ^14^C-palmitate into CO_2_, respectively.

### 2.3. Tissue Triglycerides and Cholesterol Measurements

To determine triglycerides and cholesterol in the liver, kidneys and heart, tissues were powdered under liquid N_2_ and extracted in a chloroform:methanol mixture, after which, 2% KH_2_PO_4_ was added and the solution was centrifuged. The organic phase was collected and evaporated under N_2_. The resulting pellet was dissolved in isopropyl alcohol, and lipid concentrations were determined by an enzymatic assay (Erba-Lachema, Brno, Czech Republic).

### 2.4. Biochemical Analyses

Serum levels of glucose, triglycerides, total and HDL-cholesterol were measured by commercially available kits (Erba-Lachema, Brno, Czech Republic). NEFA levels were determined using an acyl-CoA oxidase-based colorimetric kit (Roche Diagnostics GmbH, Mannheim, Germany). Serum insulin, glucagon, leptin, adiponectin, MCP-1, TNFα, and IL-6 concentrations were determined using a rat insulin ELISA kit (Mercodia, Uppsala, Sweden; MyBioSource, San Diego, CA, USA; eBioscience-Beder, Wien, Austria; BioVendor, Brno, Czech Republic). Rat serum CRP and human serum CRP were also analyzed by ELISA kits (Alpha Diagnostics International, San Antonio, CA, USA). Serum and renal β-hydroxybutyrate (BHB) concentrations were determined by a colorimetric assay kit (Sigma-Aldrich, Saint Louis, MO, USA).

### 2.5. Parameters of Oxidative Stress

Oxidative stress was evaluated according to the activities of antioxidant enzymes, concentrations of reduced and oxidized glutathione, and levels of lipoperoxidation products TBARS and conjugated dienes [20]. The activities of superoxide dismutase (SOD), glutathione peroxidase (GSH-Px), and glutathione reductase (GR) were analyzed using Cayman Chemicals assay kits (Ann Arbor, MI, USA). Catalase (CAT) activity was measured spectrophotometrically. Lipoperoxidation products were determined by assaying the reaction with thiobarbituric acid. The levels of conjugated dienes were analyzed by extraction in the media (heptane:isopropanol = 2:1) and measured spectrophotometrically in heptane’s layer. Concentrations of reduced (GSH) and oxidized (GSSG) forms of glutathione were determined using an HPLC diagnostic kit with fluorescence detection (ChromSystems, Gräfelfing, Germany).

### 2.6. Urine Collection and Microalbuminuria

Rats were placed in metabolic cages for 16 h to obtain urine samples for the analysis of urinary excretion of albumin, creatinine, sodium, and glucose. The level of albumin in urine was analyzed by HPLC method with UV-VIS detection and adjusted for creatinine concentration. Urinary glucose levels were analyzed by glucose oxidase assay (Erba-Lachema, Brno, Czech Republic). Urinary protein was measured using the Folin method with bovine serum albumin as a standard [21].

### 2.7. Cardiac Function and Blood Pressure Measurements

In a separate group of adult rats, cardiac function was evaluated at the beginning (week 0) and at the end of experiment (week 7). Gross morphology and function of the left ventricle was assessed by non-invasive ultrasound technique (transthoracic echocardiography, GE Vingmed System Seven with a 14 MHz linear matrix probe) in anesthetized rats (2% isoflurane). Within the baseline echocardiographic evaluation, the following diastolic and systolic dimensions of the left ventricle (LV) were measured: anterior wall thickness (AWTd, AWTs), posterior wall thickness (PWTd, PWTs), and left ventricular cavity diameter (LVDd, LVDs), after which the fractional shortening (FS, %) was calculated [22]. At the end of the study, blood pressure and heart rate (HR) were recorded using a pressure transducer and a multichannel recorder (AD Instruments, Bella Vista, Australia) in conscious rats that were cannulated one day prior to the experiment. Briefly, a polyethylene cannula was implanted under 2.5% isoflurane anesthesia (PE 50 for BP measurement in the left carotid artery), and exteriorized in the interscapular region.

### 2.8. Quantitative PCR (qPCR)

1 µg total RNA was used to synthesize cDNA using SuperScript IV reverse transcriptase (ThermoFisher, Waltham, MA, USA) according to the manufacturer’s protocol. The resulting cDNAs were then used as templates in quantitative real-time PCR (qPCR) reactions. Primers for qPCR reactions were designed using PrimerBLAST [23] to span at least one exon-exon junction: amplicon size was set to 70–150 bp. Each qPCR reaction contained equivalent of 8 ng of input RNA, 300 nM of each forward and reverse primer and 1× Power-up SYBRGreen master mix (ThermoFisher) and was amplified in 7900HT (Applied Biosystems, San Francisco, CA, USA). For the qPCR experiment, cycle threshold (Ct) values of selected genes were normalized relative to the expression of the peptidylprolyl isomerase A (*Ppia*, cyclophilin) gene (for kidney cortex), which served as the internal control, with results being determined in triplicates. Relative quantification was performed using the ΔΔCt method.

### 2.9. Histology

Kidneys (n = 5) from each group were cut along the longitudinal axis and processed for paraffin embedding. Multiple 4 μm thick sections were cut and stained with Hematoxylin-Eosin, PAS and Azan-Mallory trichrome stain for observation under light microscopy. Slides were observed and pictures acquired with a digitalized camera by an experienced pathologist in a blinded manner. Renal damage, as determined using the glomerulosclerosis index and tubulointerstitial injury, was examined, as described previously [24,25], using the Nikon NIS-Elements AR 3.1 morphometric program (Nikon, Tokyo, Japan).

### 2.10. Statistical Analysis

All data are expressed as means ± S.E.M. Differences between experimental groups were analyzed by two-way ANOVA with adjustments for multiple comparisons by Holm–Sidak testing. Statistical significance was defined as *p* < 0.05.

## 3. Results

### 3.1. Effects of Empagliflozin on Body Weight, Weights of Fat Depots, Cardiac Function and Blood Pressure

As can be seen in Table 1, in both age groups of transgenic SHR-CRP rats, empagliflozin administration reduced body weight as well as adiposity, as evidenced by the significantly decreased relative weights of epididymal and perirenal fat depots. Left ventricular function expressed as fractional shortening (FS) tended to be reduced in adult SHR-CRP during the study, suggesting the deterioration of heart function in these ageing rats (Table 2). Moreover, anterior and posterior diastolic left ventricle diameter (AWTd and PWTd) substantially increased in untreated SHR-CRP rats while it remained stable in empagliflozin-treated animals throughout the study. Developing concentric hypertrophy is seen also in relative wall thickness, which tended to be higher in untreated rats as compared with treated animals, which corresponds to a decrease of relative heart weight at the end of the study. Nevertheless, no effect on blood pressure or heart rate was demonstrated in either age group of empagliflozin-treated rats (Table 1).

### 3.2. Effects of Empagliflozin on Metabolic Parameters and Insulin Sensitivity

**Figure 1:**
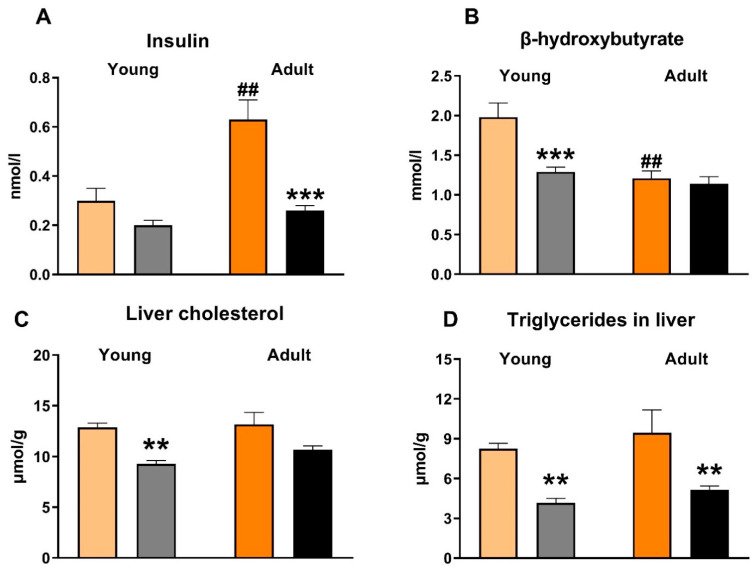
The effect of empagliflozin treatment on plasma insulin (**A**), plasma β-hydroxybutyrate (**B**), liver cholesterol (**C**) and liver triglycerides (**D**). *p* < 0.05 empagliflozin vs. untreated group, *p* < 0.05 vs. respective young group. ## denotes *p* < 0.01, ** denotes *p* < 0.01; *** denotes *p* < 0.001; Data are means ± SEM; n = 7–8 for each group.

**Figure 2:**
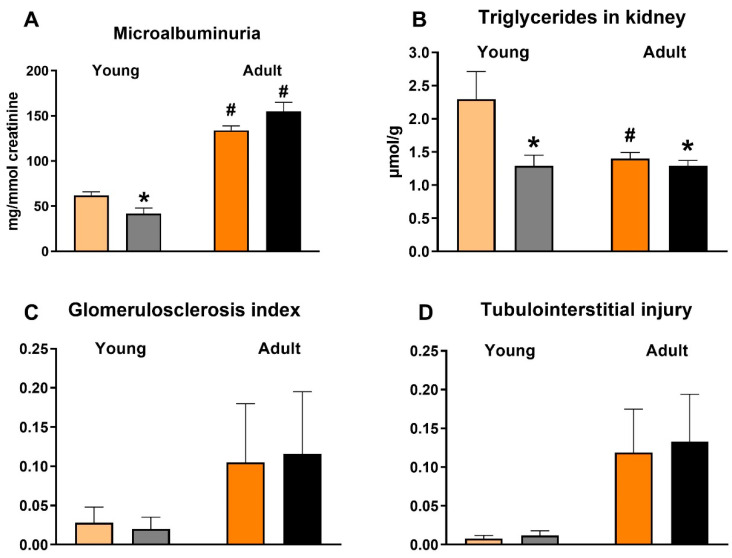
The effect of empagliflozin treatment on microalbuminuria (**A**), triglycerides in kidney (**B**), glomerulosclerosis index (**C**) and tubulointerstitial injury (**D**). * *p* < 0.05 empagliflozin vs. untreated group, # *p* < 0.05 vs. respective young group. Data are means ± SEM; n = 7–8 for each group.

Compared to untreated animals, empagliflozin-treated SHR-CRP rats of both age groups exerted no significant differences in serum lipids—triglycerides, non-esterified fatty acids (NEFA), total and HDL cholesterol (Table 3). However, these parameters were substantially affected by the ageing of the animals; adult rats showed increased levels of serum triglycerides and cholesterol compared to young rats. Both age groups of empagliflozin-treated SHR-CRP rats exhibited markedly reduced serum insulin (Figure 1), while the serum levels of fasting and non-fasting glucose were not significantly changed (Table 3). Moreover, empagliflozin treatment reduced ectopic hepatic triglyceride accumulation and hepatic cholesterol concentration (Figure 1), as well as ectopic triglyceride accumulation in kidneys (Figure 2). β-hydroxybutyrate levels were decreased following empagliflozin treatment, this effect being more prominent in young rats than in old ones (Figure 1). By contrast, there were no significant differences in the sensitivity of adipose tissue to insulin action, measured ex vivo as the incorporation of glucose into adipose tissue lipids (lipogenesis). Basal and adrenaline-stimulated lipolysis were also unchanged after empagliflozin treatment in both age groups (Table 3).

### 3.3. Effects of Empagliflozin on Inflammatory and Oxidative Stress Parameters

In adult SHR-CRP rats, empagliflozin treatment reduced markers of systemic inflammation. The levels of rat (but not human) CRP, leptin, and MCP-1 were significantly decreased in the serum of SHR-CRP rats, while other inflammatory parameters (TNFα and IL-6) were not affected (Table 4). However, when compared with adult animals, young empagliflozin-treated SHR-CRP rats had decreased serum levels of leptin and human CRP. As shown in Table 5, there was a substantial augmentation of oxidative stress in the kidney cortex of aged SHR-CRP rats. The empagliflozin treatment attenuated oxidative stress parameters, most of these effects being more pronounced in young than in adult rats. The lipoperoxidation products TBARS and conjugated dienes (CD) were significantly decreased and the activities of antioxidant enzymes (glutathione peroxidase, catalase) and the concentration of reduced glutathione were increased.

### 3.4. Effects of Empagliflozin on Renal Expression of Selected Pro-Inflammatory Genes

**Figure 3:**
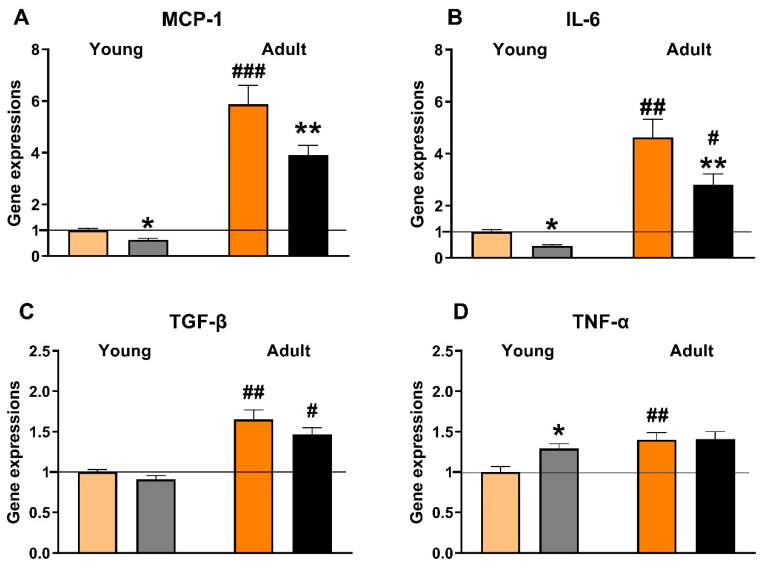
Relative mRNA expression of selected pro-inflammatory genes in the kidneys—monocyte chemoattractant protein-1 (MCP-1; (**A**)), Interleukin-6 (IL-6; (**B**)), Transforming growth factor-β (TGF-β; (**C**)), and Tumor necrosis factor-α (TNF-α; (**D**)). # *p* < 0.05 vs. respective young group. * denotes *p* < 0.05; ** denotes *p* < 0.01; ## denotes *p* < 0.01; ### denotes *p* < 0.001. Data are means ± SEM; n = 7–8 for each group.

The changes in renal expression of selected genes (*Ccl2*, *Il6*, *Tgfb*, *Tnf*) indicated that ageing was related to the substantial aggravation of inflammation in SHR-CRP rats (Figure 3). In addition, empagliflozin treatment reduced the expression of *Ccl2* (to 63% in young and to 66% in adult rats) and *Il6* (to 47% in young and to 61% in adult rats) genes in both age groups, while the expression of *Tgfb* and *Tnf* genes was not affected.

### 3.5. Effects of Empagliflozin on Renal Function Markers and Histological Analysis

**Figure 4:**
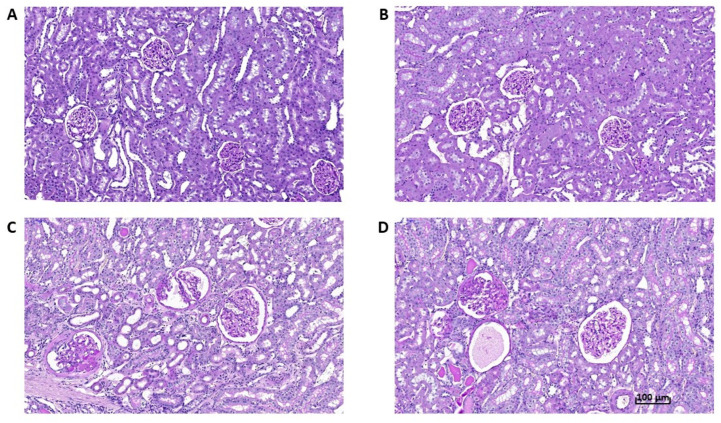
Representative histological images of renal cortex (PAS, 200×) (PAS, original objective 20×) of young (**A**,**B**) and adult (**C**,**D**) untreated (**A**,**C**) and empagliflozin-treated SHR-CRP rats (**B**,**D**). A scale bar is shown in Figure 4D.

In both age groups, urinary glucose excretion was more than twenty times higher, and sodium excretion two times higher, in empagliflozin-treated SHR-CRP rats (data not shown), suggesting an effective blockade of the SGLT-2 transporter. Microalbuminuria was substantially aggravated in adult SHR-CRP rats as compared with young animals, which is in accordance with the established phase of the incipient nephropathy associated with transgenic CRP expression at the age of one year. This was also confirmed by slightly increased histological markers found in old rats (Figure 2 and Figure 4) and also supported by increased gene expression levels of pro-inflammatory markers MCP-1 and IL-6 (Figure 3). Unexpectedly, empagliflozin treatment decreased microalbuminuria only in young, but not in adult animals. However, this was not paralleled by a decrease of glomerulosclerosis index or tubulointerstitial injury index. Both control and empagliflozin-treated adult SHR-CRP rats exhibited moderate changes with focal segmental glomerular sclerosis accompanied by smaller areas of tubular atrophy when compared to empagliflozin-treated rats. Contrary to adult rats, young SHR-CRP rats exhibited substantially reduced histopathological changes in the kidney and showed no effects of empagliflozin treatment (Figure 4).

## 4. Discussion

### 4.1. Renoprotective Effects of Empagliflozin Are Associated with Reduced Ectopic Fat Accumulation and Lower Inflammation and Oxidative Stress

The results of the current study demonstrated the significant renoprotective effects of empagliflozin in SHR-CRP rats. Specifically, reduced ectopic fat accumulation in the kidney was associated with decreased inflammation and oxidative stress and reduced microalbuminuria in young rats. Moreover, we confirmed that in the spontaneously hypertensive rats expressing human C-reactive protein (a model of metabolic syndrome, inflammation and organ damage), there is an age-dependent increase of insulin resistance, inflammatory markers in kidneys (MCP-1, TGF-β, TNF-α and IL-6), and deterioration of kidney function [19].

The reduced weight of renal fat following empagliflozin treatment was also associated with a reduced tissue expression of genes coding for enzymes that regulate inflammation (MCP-1 and IL-6) in both young and adult rats, which is consistent with the reduced serum levels of these pro-inflammatory markers. Conversely, the effects of empagliflozin treatment on gene expression of other pro-inflammatory cytokines TGF-β, TNF-α were not demonstrated. However, we and others found ambiguous effects of gliflozins therapy on distinct pro-inflammatory markers in non-diabetic rat strains—sometimes affecting more TNF-α [17], MCP-1 [18], or IL-1β and NF-κB [18]. Moreover, it could not be excluded that only those pro-inflammatory parameters which were upregulated by the insertion of the CRP transgene (IL-6, but not TNF-α) [19], could be influenced by empagliflozin therapy. The higher albuminuria found in adult control and treated animals is compatible with the long-term effect of high blood pressure, as well as the pro-hypertensive effect of CRP transgene in these animals. Thus, the absence of an empagliflozin effect on the reduction of albuminuria in adult rats could be explained either by the existing long-term changes in the kidneys of ageing animals or by the higher susceptibility of young animals to pharmacological interventions [26]. Similarly, our previous study performed in another non-diabetic hypertensive model—adult Ren-2 transgenic rats—did not show any effect of empagliflozin on proteinuria or albuminuria [17]; while a reduction of albuminuria was disclosed in a pre-diabetic rat model—hereditary hypertriglyceridemic rats [18]. Conversely, a reduction of albuminuria in young rats was associated with substantial attenuation of several parameters of oxidative stress (reduced levels of lipoperoxidation products TBARS and conjugated dienes, increased activity of glutathione peroxidase, increased glutathione levels, etc.) suggesting that intervention applied at an early age (critical developmental window) could be more effective [26]. Altogether, these results support the theory that empagliflozin treatment protects against the incipient nephropathy associated with the overexpression of the CRP transgene by reducing oxidative stress and inflammation either directly or as a consequence of reduced ectopic fat accumulation in the kidney.

In fact, in several animal models, including streptozotocin-induced diabetic mice and rats [27,28], Akita mice [29], OVE26 mice [30], and db/db mice [12,30], it was reported that kidney disease was associated with lipid accumulation and increased activity of pro-inflammatory cytokines, resulting in albuminuria, glomerular mesangial expansion, and tubulointerstitial fibrosis. Ectopic fat accumulation was observed in kidney biopsies of humans with type 2 diabetes mellitus [31,32]. Recently, Wang et al. [12] reported that SGLT-2 inhibition by JNJ-39933673 in db/db mice was associated with decreased renal lipid accumulation and prevention of the development of nephropathy. In addition, Hosokawa et al. [33] showed that ipragliflozin decreased ectopic lipid accumulation in tubular cells in diabetic mice. Although further studies are needed, it is plausible that, in addition to the well-known anti-inflammatory, anti-proliferative, and anti-fibrotic effects of SGLT-2 inhibitors, the reduction of tubular lipid deposition could contribute to the renoprotective mechanism of these molecules [34].

The attenuation of renal inflammation following empagliflozin treatment can lead to a decreased permeability of endothelial cells and subsequent alterations in the hemodynamics of the kidney [35], contributing to the improvement of renal function. The evidence for the anti-inflammatory potential of SGLT-2 inhibitors has been previously demonstrated in diabetic animal models [36]. Moreover, in human proximal tubular cells, SGLT-2 inhibitors (tofogliflozin) attenuated the expression of pro-inflammatory markers [37]. In diabetic patients, SGLT-2 inhibitors were found to reduce systemic levels of pro-inflammatory markers IL-6 and TNFα [8]. The SGLT-2 inhibitor-mediated anti-inflammatory effects, however, have not been shown under normoglycemic conditions.

Consistent with its anti-inflammatory properties, empagliflozin also attenuated oxidative stress—another key pathway known to cause kidney impairment. In the present study, empagliflozin markedly improved renal oxidative stress with the effect also being more pronounced in young rats. Empagliflozin regulates oxidative stress in the kidney cortex by stimulating antioxidant enzyme activity GSH-Px and catalase via the upregulation of Nrf2 rather than the direct inactivation of free radicals. In addition, increased GSH-Px activity can play a role in decreasing lipid peroxidation by participating in the removal of lipoperoxidation products. In the kidney cortex, increased GSH-Px activity following empagliflozin treatment was also linked to increased glutathione levels, a sensitive marker of oxidative damage. A markedly alleviated renal oxidative stress can be one of the pleiotropic metabolic mechanisms of empagliflozin that can also contribute to the improvement of renal function.

### 4.2. Effects of Empagliflozin on Insulin, β-Hydroxybutyrate, NEFA and Leptin Concentrations

In our study, empagliflozin markedly reduced hyperinsulinemia, although no significant changes in insulin sensitivity were observed in muscle and adipose tissue, as well as in fasting or non-fasting glucose levels. Thus, other factors contributing to the improved insulin sensitivity following empagliflozin treatment such as decreased NEFA and leptin levels can play a role. Although Nishimura et al. [38] have shown that SGLT-2 inhibitors promote systemic NEFA mobilization associated with the induction of ketone bodies as alternative substrate for energy metabolism, we observed no increase of NEFA or β-hydroxybutyrate (BHB) after empagliflozin administration. On the contrary, empagliflozin even reduced circulating BHB concentration. A number of possible mechanisms of SGLT-2 inhibitors are implicated in their cardioprotective effects, which go beyond their diuretic and antihyperglycemic effects. The increase of ketone bodies is only one of the proposed mechanisms, which may not always apply. In addition, increased ketone bodies after SGLT-2 inhibitors are rather related to systemic alterations in substrate utilization and reduced glucose oxidation in preference for fatty acid oxidation. Thus, empagliflozin in a non-diabetic model with genetic hypertension and chronic inflammation affects heart function by other mechanisms than ketone bodies utilization. In our previous study with prediabetic animal model with vascular complications, we observed increased BHB in serum and the heart, but not increased BHB utilization in the heart [20]. In a study with diabetic obese rats, Abdurrachim et al. [39] reported that empagliflozin decreased ketone bodies utilization in the heart despite increasing circulating levels of BHB.

The markedly decreased circulating leptin levels observed in this study may not only contribute to improved insulin resistance, but may also alleviate inflammation and cardiovascular damage. In addition to the effect on insulin sensitivity, leptin may have a pathophysiological role in sodium regulation, as well as in cardiac and renal inflammation and fibrosis [40]. Circulating leptin levels presage the development of heart failure in elderly people and a decline in the glomerular filtration rate in longitudinal studies [41,42]. Although the mechanism/s of leptin action in heart failure are not fully understood, one potential mechanism might be its effect on epicardial adipose expansion and on calcium handling in cardiomyocytes leading to impaired myocardial relaxation. SGLT-2 inhibitors lead to decreases in serum aldosterone, reduction in the activity of NHE1 (sodium-hydrogen exchanger isoform 1), and can probably directly suppress leptin secretion and its paracrine actions on the heart and kidneys to promote fibrosis [40]. All these effects may underlie the action of SGLT-2 inhibitors to ameliorate cardiac and renal injury. Although a positive effect on plasma leptin levels has been observed in other studies with empagliflozin [43,44], it is unclear whether empagliflozin action directly impacts on adipose tissue function or whether it is associated with visceral fat loss. Leptin is secreted by epicardial and perirenal adipose tissue, thus the reduction of these fat depots can also contribute to decreased leptin levels. Although adipose tissue insulin sensitivity was not affected, empagliflozin treatment reduced the weight of visceral adipose tissue, and it is possible that reduced adiposity positively influenced secretion of other adipocytokines, in addition to leptin.

### 4.3. Effects of Empagliflozin on Liver Triglycerides and Cholesterol

Although circulating lipids were not affected in the present study, empagliflozin treatment significantly decreased hepatic triglycerides, as well as cholesterol content. According to our previous studies, empagliflozin modulates genes related to lipid synthesis and fatty acid metabolism, while it has no effect on genes involved in lipid oxidation and transport [20]. Thus, we speculate that reduced lipid accumulation in the liver is probably associated with the inhibited lipogenesis. The suppression of SCD1—the main lipogenic enzyme—was observed in our [20], as well as in another animal study with obese mice [45]. The reduced ectopic hepatic lipid deposition can also ameliorate insulin resistance. The intrahepatic accumulation of fatty acids and lipotoxic intermediates interferes with intracellular signaling pathways like insulin signaling, and can induce endoplasmic reticulum stress; both can contribute to the development of insulin resistance [46]. The hepatic lipid accumulation and impaired lipid metabolism in the liver are independent risk factors for cardiovascular events, thus, the reduced hepatic lipid accumulation can contribute to the cardioprotective effect of empagliflozin.

## 5. Conclusions

It can be concluded that treatment of SHR-CRP rats with empagliflozin is associated with reduced renal lipid accumulation, inflammation, and oxidative stress, resulting in the attenuation of renal damage; these beneficial effects being more pronounced in young rats. By contrast, the metabolic effects of empagliflozin prevailed in adult rats.

## Figures and Tables

**Table 1:** Basal parameters in experimental groups during the study.

	Young		Adult				
	Control	Empa	Control	Empa	P_AGE_	P_TREATMENT_	P_INTER-ACTION_
Body weight (g)	347 ± 6	316 ± 7 **	379 ± 6 ^#^	368 ± 5 ^#^	<0.001	<0.01	n.s.
Epididymal fat (g/100 g BW)	0.58 ± 0.02	0.56 ± 0.01	0.52 ± 0.03	0.39 ± 0.02 ***	<0.001	<0.01	n.s.
Perirenal fat (g/100 g BW)	0.43 ± 0.04	0.33 ± 0.01	0.57 ± 0.06	0.41 ± 0.03 *	<0.01	<0.01	n.s.
Liver weight (g/100 g BW)	3.05 ± 0.11	3.30 ± 0.04 *	4.03 ± 0.09 ^#^	3.68 ± 0.04 **^#^	<0.001	n.s.	<0.001
Heart weight (g/100 g BW)	0.36 ± 0.01	0.34 ± 0.01	0.41 ± 0.01 ^#^	0.39 ± 0.01 *^#^	<0.001	<0.05	n.s.
Kidney weight (g/100 g BW)	0.66 ± 0.03	0.70 ± 0.01	0.74 ± 0.01	0.75 ± 0.01	<0.01	n.s.	n.s.
Mean arterial pressure (mm Hg)	192 ± 11	197 ± 7	190 ± 4	197 ± 6	n.s.	n.s.	n.s.
Heart rate (bpm)	373 ± 8	380 ± 5	377 ± 8	355 ± 8 ^#^	n.s.	n.s.	n.s.

**^# ^**P_AGE_ denotes the significance of young vs. adult SHR-CRP rats (age effect); P_TREATMENT_ denotes the significance of empagliflozin treatment (treatment effects); P_INTERACTION_ denotes the significance of different empagliflozin effects in various strains (treatment vs. strain comparison). For multiple comparisons (empagliflozin treatment vs. non-treated controls) Fisher’s LSD post-hoc test was used; * denotes *p* < 0.05; ** denotes *p* < 0.01; *** denotes *p* < 0.001; n.s. denotes not significant, Data are means ± SEM; n = 7–8 for each group.

**Table 2:** Echocardiography parameters in adult animals at the end of the study.

	SHR-CRP			
	Baseline		End of Study	
	Control	Empagliflozin	Control	Empagliflozin
AWTd (mm)	1.96 ± 0.02	1.93 ± 0.03	2.14 ± 0.03 ^@^	1.96 ± 0.04 *
LVDd (mm)	7.75 ± 0.14	7.20 ± 0.09	7.72 ± 0.11	7.41 ± 0.27
PWTd (mm)	1.95 ± 0.02	1.95 ± 0.03	2.08 ± 0.02 ^@^	1.92 ± 0.05 *
AWTs (mm)	2.87 ± 0.06	2.86 ± 0.06	2.99 ± 0.06 ^@^	2.80 ± 0.04 *
LVDs (mm)	4.75 ± 0.09	4.79 ± 0.07	4.35 ± 0.05	4.58 ± 0.20
PWTs (mm)	2.87 ± 0.04	2.88 ± 0.06	3.00 ± 0.04 ^@^	2.80 ± 0.04 *
FS (%)	39.1 ± 0.47	39.5 ± 0.26	37.8 ± 0.31	38.3 ± 0.53
HR (bpm)	329 ± 9	334 ± 5	316 ± 9	306 ± 11

SHR-CRP—spontaneously hypertensive rat (SHR) expressing human C-reactive protein (CRP), AWTd—anterior wall thickness diastolic, LVDd—left ventricular diameter diastolic, PWTd—posterior wall thickness diastolic, AWTs—anterior wall thickness systolic, LVDs—left ventricular diameter systolic, PWTs—posterior wall thickness systolic, FS—fractional shortening, HR—heart rate, * *p* < 0.05 empagliflozin vs. control SHR-CRP, **^@^** *p* < 0.05 vs. baseline.

**Table 3:** Biochemical parameters in the experimental groups at the end of the study.

	Young		Adult				
	Control	Empagliflozin	Control	Empagliflozin	P_AGE_	P_TREATMENT_	P_INTER_ _-ACTION_
Triglycerides (mmol/L)	0.35 ± 0.04	0.39 ± 0.04	0.72 ± 0.13 ^#^	0.57 ± 0.04 ^#^	<0.01	n.s.	n.s.
Total cholesterol (mmol/L)	1.24 ± 0.19	1.00 ± 0.03	1.33 ± 0.08	1.50 ± 0.05 ^#^	<0.01	n.s.	n.s.
HDL-cholesterol (mmol/L)	1.06 ± 0.13	0.85 ± 0.01 *	1.06 ± 0.05	1.22 ± 0.02 ^#^	<0.05	n.s.	<0.05
NEFA (mmol/L)	0.51 ± 0.02	0.44 ± 0.01 *	0.44 ± 0.02	0.47 ± 0.02	n.s.	n.s.	<0.05
Fasting glucose (mmol/L)	4.3 ± 0.1	4.3 ± 0 1	4.6 ± 0.1	4.5 ± 0.2	n.s.	n.s.	n.s.
Non-fasting glucose (mmol/L)	7.1 ± 0.2	6.8 ± 0.1	7.4 ± 0.3	6.9 ± 0.3	n.s.	n.s.	n.s.
AUC0–120 (OGT test)	842 ± 19	802 ± 11	842 ± 25	905 ± 63	n.s.	n.s.	n.s.
Lipogenesis 0 (nmol/g/2 h)	1397 ± 161	1582 ± 204	1105 ± 82	1206 ± 92	<0.05	n.s.	n.s.
Lipogenesis 250 (nmol/g/2 h)	2007 ± 106	2372 ± 350	1948 ± 313	1705 ± 232	<0.05	n.s.	n.s.
Lipolysis 0 (nmol/g/2 h)	4.70 ± 0.24	3.84 ± 0.32	4.61 ± 0.54	4.78 ± 0.49	n.s.	n.s.	n.s.
Lipolysis 250 (nmol/g/2 h)	6.52 ± 0.34	5.28 ± 0.76	8.06 ± 0.76	8.36 ± 0.98 ^#^	n.s.	n.s.	n.s.
Glycogenesis 0 (nmol/g/2 h)	1366 ± 240	1505 ± 307	1803 ± 128	1843 ± 243	<0.01	n.s.	n.s.
Glycogenesis 250 (nmol/g/2 h)	1642 ± 248	2522 ± 462	1995 ± 214	2059 ± 211	n.s.	n.s.	n.s.

**^# ^**P_AGE_ denotes the significance of young vs. adult SHR-CRP rats (age effect); P_TREATMENT_ denotes the significance of empagliflozin treatment (treatment effects); Interaction denotes the significance of empagliflozin in various strains (treatment vs. strain comparison). For multiple comparisons (empagliflozin treatment vs. non-treated controls) Fisher´s LSD post-hoc test was used; * denotes *p* < 0.05; n.s. denotes not significant, Data are means ± SEM; n = 7–8 for each group. AUC—area under curve from the oral glucose tolerance (OGT) test.

**Table 4:** Inflammatory parameters in serum in experimental groups at the end of the study.

	Young		Adult				
	Control	Empagliflozin	Control	Empagliflozin	P_AGE_	P_TREATMENT_	P_INTER-_ _ACTION_
hs CRP human (mg/mL)	299 ± 59	221 ± 5 *	201 ± 9	227 ± 10	n.s.	n.s.	<0.05
hs CRP rat (mg/mL)	0.82 ± 0.15	0.51 ± 0.15	1.95 ± 0.10 ^#^	1.42 ± 0.13 *^#^	<0.001	<0.01	n.s.
MCP-1 (ng/mL)	4.36 ± 0.67	3.53 ± 0.17	9.47 ± 0.52 ^#^	7.43 ± 0.17 **^#^	<0.001	<0.01	n.s.
IL-6 (pg/mL)	51.86 ± 23.17	50.62 ± 17.96	49.75 ± 15.86	50.26 ± 17.23	n.s.	n.s.	n.s.
Leptin (ng/mL)	2.39 ± 0.39	0.73 ± 0.23 ***	1.35 ± 0.03	1.23 ± 0.02	n.s.	<0.001	<0.01

**^# ^**P_AGE_ denotes the significance of young vs. adult SHR-CRP rats (age effect); P_TREATMENT_ denotes the significance of empagliflozin treatment (treatment effects); Interaction denotes the significance of empagliflozin in various strains (treatment vs. strain comparison). For multiple comparisons (empagliflozin treatment vs. non-treated controls) Fisher´s LSD post-hoc test was used; * denotes *p* < 0.05; ** denotes *p* < 0.01; *** denotes *p* < 0.001; n.s. denotes not significant, Data are means ± SEM; n = 7–8 for each group. CRP—C-reactive protein, MCP-1—Monocyte chemoattractant protein-1, IL-6—Interleukin-6.

**Table 5:** Oxidative stress parameters in kidney cortex in experimental groups at the end of the study.

	Young		Adult				
	Control	Empagliflozin	Control	Empagliflozin	P_AGE_	P_TREATMENT_	P_INTER-_ _ACTION_
SOD	0.045 ± 0.003	0.048 ± 0.003	0.046 ± 0.003	0.050 ± 0.002	n.s.	n.s.	n.s.
GSH-Px	148 ± 14	199 ± 12 **	163 ± 14	214 ± 8 **	n.s.	<0.001	n.s.
GR	73 ± 5	67 ± 7	78 ± 4	86 ± 3 ^#^	<0.05	n.s.	n.s.
CAT	626 ± 33	790 ± 32 ***	553 ± 21	636 ± 30 *^#^	<0.001	<0.001	n.s.
CD	21.7 ± 1.2	17.0 ± 1.8 *	22.6 ± 1.0	16.6 ± 1.2 **	n.s.	<0.001	n.s.
TBARS	0.78 ± 0.03	0.50 ± 0.03 ***	0.65 ± 0.02	0.52 ± 0.03 **	n.s.	<0.001	<0.05
GSH	25.8 ± 5.3	37.8 ± 4.3 ***	13.7 ± 0.7 ^#^	14.3 ± 0.1 ^#^	<0.001	<0.001	<0.001
GSSG	2.4 ± 0.1	2.7 ± 0.1	1.8 ± 0.1 ^#^	1.8 ± 0.1 ^#^	<0.01	n.s.	n.s.
GSH/GSSG	12.1 ± 0.8	14.3 ± 0.6	7.9 ± 0.4 ^#^	7.9 ± 0.2 ^#^	<0.001	n.s.	n.s.

**^# ^**P_AGE_ denotes the significance of young vs. adult SHR-CRP rats (age effect); P_TREATMENT_ denotes the significance of empagliflozin treatment (treatment effects); P_INTERACTION_ denotes the significance of empagliflozin in various strains (treatment vs. strain comparison). For multiple comparisons (empagliflozin treatment vs. non-treated controls) Fisher´s LSD post-hoc test was used; * denotes *p* < 0.05; ** denotes *p* < 0.01; *** denotes *p* < 0.001; n.s. denotes not significant, Data are means ± SEM; n = 7–8 for each group. SOD—superoxide dismutase (U/mg), GSH-Px–glutathione peroxidase (µmol/NADPH/min/mg), GR–glutathione reductase (µmol/NADPH/min/mg), CAT—catalase (µmol/H_2_O_2_/min/mg), CD—conjugated dienes (nmol/mg), TBARS—thiobarbituric acid reactive substances (µmol/g), GSH—reduced glutathione (µmol/g), GSSG—oxidized glutathione (µmol/g).

## Data Availability

All data arising from this study are contained within the article.
